# Melatonin and N- Acetylcysteine as Remedies for Tramadol-Induced Hepatotoxicity in Albino Rats

**DOI:** 10.15171/apb.2017.044

**Published:** 2017-09-25

**Authors:** Elias Adikwu, Bonsome Bokolo

**Affiliations:** ^1^Department of Pharmacology, Faculty of Basic Medical Sciences, University of Port Harcourt, Choba, Rivers State, Nigeria.; ^2^Department of Pharmacology, Faculty of Basic Medical Sciences, Niger Delta University Wilberforce Island, Bayelsa State, Nigeria.

**Keywords:** Tramadol, Liver, Toxicity, Antioxidants, Pretreatment, Rat

## Abstract

***Purpose:*** The therapeutic benefit derived from the clinical use of tramadol (TD) has been characterized by hepatotoxicity due to misuse and abuse. The implications of drug-induced hepatotoxicity include socio-economic burden which makes the search for remedy highly imperative. The present study investigated the protective effects of melatonin (MT) and n-acetylcysteine (NAC) on TD-induced hepatotoxicity in albino rats.

***Methods:*** Forty five adult rats used for this study were divided into nine groups of five rats each. The rats were pretreated with 10mg/kg/day of NAC, 10mg/kg/day of MT and combined doses of NAC and MT prior to the administration of 15 mg/kg/day of TD intraperitoneally for 7 days respectively. At the termination of drug administration, rats were weighed, sacrificed, and serum was extracted and evaluated for liver function parameters. The liver was harvested, weighed and evaluated for oxidative stress indices and liver enzymes.

***Results:*** Alanine aminotransferase, alkaline phosphatase, aspartate aminotransferase, total bilirubin, conjugated bilirubin, and malondialdehyde levels were significantly (P<0.05) increased in rats administered with TD when compared to control. Furthermore, glutathione, superoxide dismutase and catalase levels were decreased significantly (P<0.05) in rats administered with TD when compared to control. The Liver of TD-treated rats showed necrosis of hepatocytes. However, the observed biochemical and liver histological alterations in TD-treated rats were attenuated in NAC and MT pretreated rats. Interestingly, pretreatment with combined doses of NAC and MT produced significant (P<0.05) effects on all evaluated parameters in comparison to their individual doses.

***Conclusion:*** Based on the findings in this study, melatonin and n- acetylcysteine could be used clinically as remedies for tramadol associated hepatotoxity.

## Introduction

Tramadol (TD) is a centrally acting opioid analgesic which is mainly used for the treatment of moderate to severe pain.^1^ Its efficiency and potency ranges between weak opioids and morphine.^2^ Clinically, hepatotoxity marked by cholelithiasis, cholecystitis, and abnormal liver function tests could occur in more than 1% of patients administered with TD.^3^ However, due to its opiate-like and analgesic effects,^4^ TD abuse, dependence as well as acute overdose have led to reported cases of hepatotoxicity and even death in humans.^5-8^ Studies in animals have reported hepatotoxicity characterized by altered levels of liver function biomarkers^9,10^ and histological damage.^11-13^ In addition, oxidative stress could be involved in TD-induced hepatotoxicity due to decrease in antioxidant defence and lipid peroxidation observed in treated animals.^14^

N-acetylcysteine (NAC) is a thiol containing molecule that is produced from amino acid cysteine joined to an acetyl group. It is a small molecule which can be easily filtered and has prompt access to intracellular compartments.^15^ Studies have shown that it is a source of sulfhydryl groups and is converted in the body to metabolites capable of stimulating glutathione synthesis, promoting detoxification, and acting directly as a free radical scavenger.^16,17^ NAC also modulates inflammatory response through signaling pathways that control pro-inflammatory nuclear factor (NF)-κB activation.^18,19^ It has a diversity of applications, which include inhibition of xenobiotic-induced toxicities largely because of the chemical properties of the thiol moiety present in its structure. Reports have shown that NAC treatment protects against acetaminophen associated hepatotoxicity in patients,^20^ in carbon tetrachloride associated hepatotoxicity in humans,^21^ and in experimental animal-induced hepatotoxicity.^22-24^

Melatonin (MT) is the major hormone of the pineal gland, but it has been detected in many other tissues. It is a highly lipophilic and hydrophilic molecule that crosses cell membranes and easily reaches subcellular compartments including mitochondria, where it seems to accumulate in high concentrations.^25^ Studies have shown that MT could regulate a variety of physiological processes which include endocrine rhythms,^26^ reproductive cycle,^27^ immunomudulatory and vasomotor effects.^28^ MT is able to prevent oxidative stress through its free radical scavenging effect and by directly increasing other antioxidant activities.^29^ Furthermore, several MT metabolites which are formed when it neutralizes damaging reactants are themselves free radical scavengers.^30,31^ MT is effective against pathological states characterized by an increase in basal rate of reactive oxygen species (ROS) production, and protects liver from oxidative damage in multiple conditions.^32^ In view of the above information this study was aimed at investigating the effects of NAC and MT on TD-induced hepatotoxicity in albino rats.

## Materials and Methods

### Animals 

Forty five adult male albino rats, weighing 250±5 g, were used for this study. Rats were housed under continuous observation in appropriate cages at room temperature with a 12-12 h light-dark cycle. The rats were housed five per cage, and fed with commercial standard diet and water *ad libitum.*

### Drugs and experimental protocol

Tramadol hydrochloride (TD) used for this study was manufactured by Zahidi Enterprise Mumbai India, while NAC and MT were obtained from Shijiazhuang AO Pharm Import and Export Co Ltd China. All other chemicals used for this study are of analytical grade. TD, (15 mg/kg/day),^33^ MT (10mg/kg/day)^34^ and NAC (10mg/kg/day)^35^ were used for this study. MT was dissolved in 0.1% ethanol and diluted with normal saline.^36,37^ Rats were divided into nine (9) groups’ 1- IX of five (5) rats each. Rats in group I and II served as placebo and solvent control and were treated intraperitoneally with 0.1% of ethanol and normal saline respectively. Rats in groups III-VI were treated with 15 mg/kg/day of TD, 10mg/kg/day of NAC, 10mg/kg/day MT, and a combination of NAC and MT intraperitoneally for 7 days respectively. Rats in group VII-IX were pretreated with MT, NAC, and combined doses of MT and NAC prior to treatment with TD intraperitoneally for 7 days respectively.

### Collection of sample

Rats were sacrificed with diethyl ether; blood was collected via cardiac puncture in anon-heparinized sample container and allowed to clot. It was centrifuged at 1500 rpm for 15 minutes and serum extracted and evaluated for biochemical parameters. The liver was surgically removed weighed and placed in iced beakers. The liver was washed in ice cold KCl solution (1.15% w/v) and then homogenized with 0.1M phosphate buffer (pH 7.2). The homogenate was centrifuged at 15000 rpm for 20 min and evaluated for liver enzymes and oxidative stress indices.

### Evaluation of biochemical parameters

Alkaline phosphatase was evaluated as reported by Babson *et al.,* 1966^38^ while aspartate aminotransferase and alanine aminotransferase were evaluated as reported by Reitman and Frankel, 1957.^39^ Serum conjugated (CB) and total bilirubin (TB) levels were evaluated as reported by Doumas et al., 1979.^40^ Superoxide dismutase was evaluated as described by Sun and Zigman, 1978,^41^ while catalase was evaluated as reported by Aebi, 1984.^42^ Reduced glutathione was assayed according to Sedlak and Lindsay, 1986,^43^ while malondialdehyde was evaluated as reported by Buege and Aust,1978.^44^

## Results and Discussion

**Figure 1:**
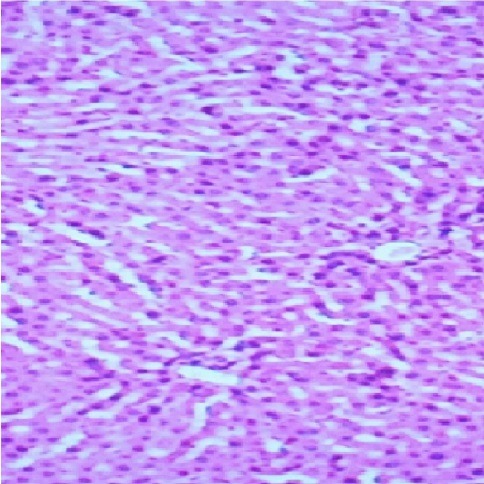
Micrograph of the control liver of rat treated with normal saline for 7 days showing normal liver architecture (Hand E X 200)

**Figure 2:**
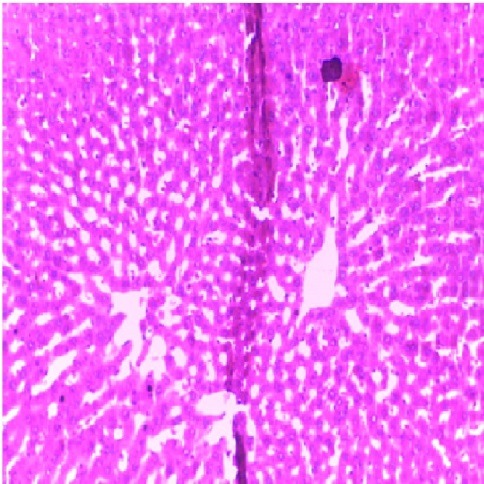
Micrograph of the liver of rat treated intraperitoneally with 10 mg/kg/day of NAC for 7 days showing normal architecture (Hand E X 200)

**Figure 3:**
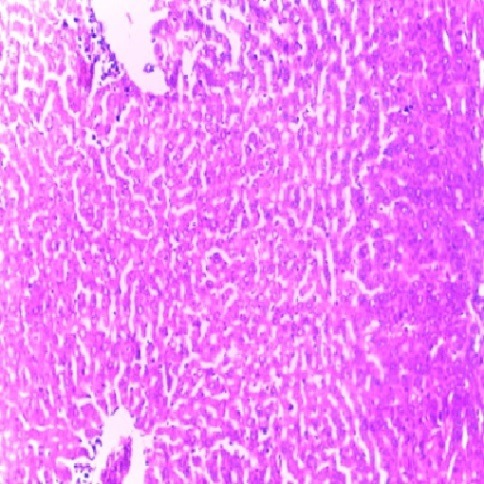
Micrograph of the liver of rat treated intraperitoneally with 10 mg/kg/day of MT for 7 days showing normal architecture (Hand E X 200)

**Figure 4:**
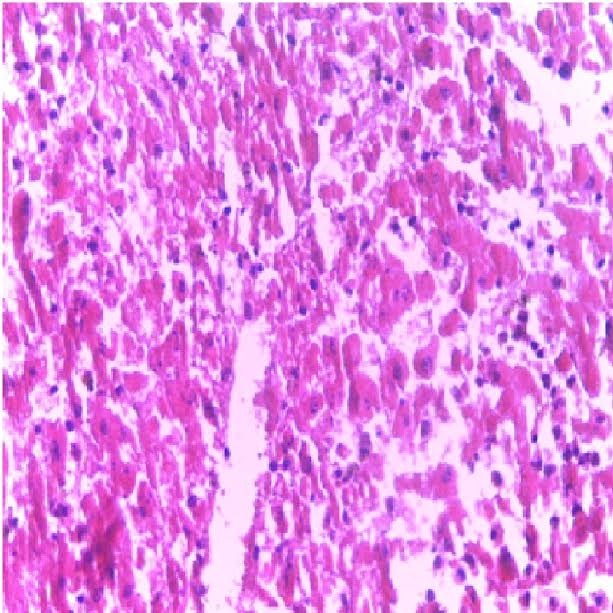
Micrograph of the liver of rat treated with 15 mg/kg/day of TD for 7 days showing hepatocytes necrosis (Hand E X 200)

**Figure 5:**
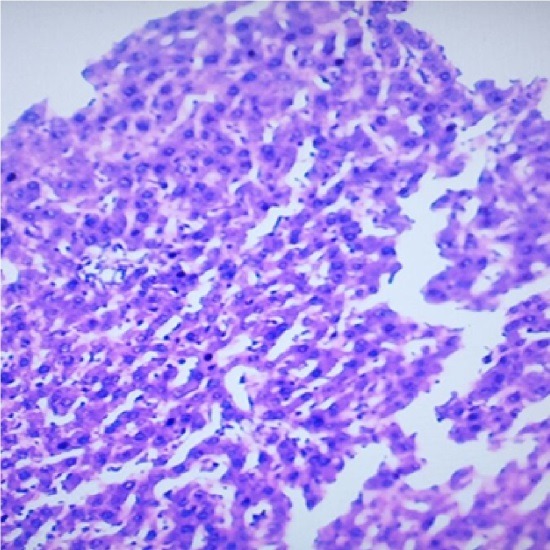
Micrograph of the liver of rat treated with 15 mg/kg/day of TD and10 mg/kg/day of NAC for 7 days showing normal architecture (Hand E X 200)

**Figure 6:**
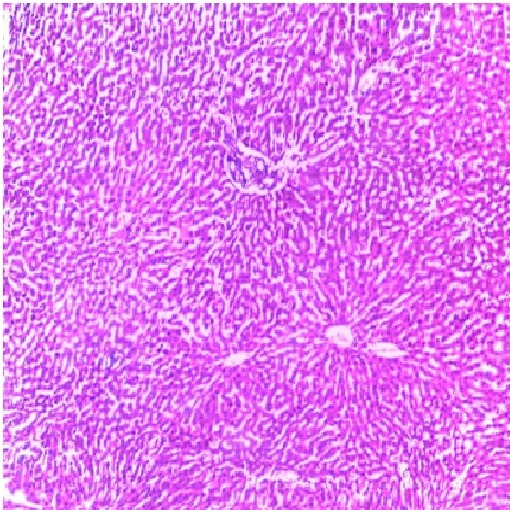
Micrograph of the liver of rat treated with 15 mg/kg/day of TD and10mg/kg/day of MT for 7 days showing normal architecture (Hand E X 200)

**Figure 7:**
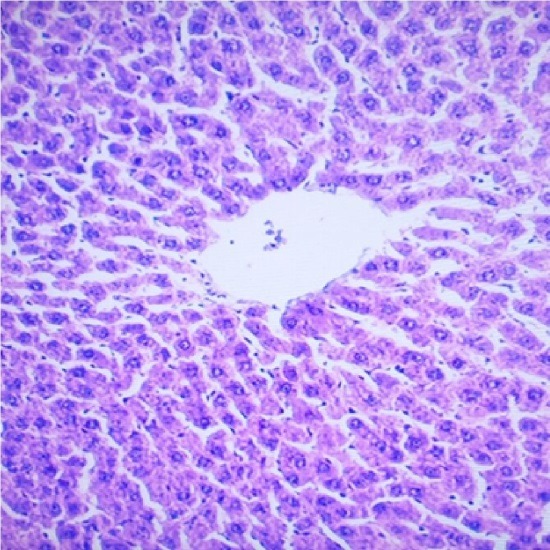
Micrograph of the liver of rat treated with 15 mg/kg/day of TD, 10mg/kg/day of NAC and 10mg/kg/day of MT for 7 days showing normal architecture (Hand E X200)

Liver is a key organ actively involved in numerous metabolic and detoxifying functions. Consequently, it continuous exposure to high levels of endogenous and exogenous oxidants which are the by-products of many biochemical pathways could lead to hepatotoxicity.^45,46^ Oxidative stress has been reported as one of the possible mechanisms of xenobiotic-induced hepatotoxicity.^47^ The present study evaluated the effects of n-acetylcysteine (NAC) and melatonin (MT) on TD -induced hepatotoxicity in albino rats. The present study did not observe significant (p>0.05) changes in body and relative liver weights of rats treated with these agents when compared to control (Table 1). The levels of AST, ALP, ALT, TB, CB and MDA were decreased whereas the levels of SOD, CAT and GSH were increased in MT and NAC treated rats. However, effects on these parameters were not significantly (p>0.05) different when compared to control (Table1 and 2). These observations are consistent with previous reports.^48-50^ On the contrary, levels of AST, ALP, ALT, TB, CB and MDA were increased significantly (p<0.05) whereas SOD, CAT and GSH levels were decreased significantly (p<0.05) in TD-treated rats in comparison to control (Table 3-5).Similar observations have been reported in previous studies.^51,52^ The microscopic examination of the liver of NAC and MT-treated rats showed normal architecture; however, the liver of TD- treated rats showed necrosis of hepatocytes (Figure 1-4). The observed histological alterations in the liver of TD-treated rats are in conjunction with earlier findings.^53^ The increases in AST, ALP, ALT, TB, and CB levels and necrosis of hepatocytes in TD-treated rats are indicators of hepatotoxicity.^53,54^ The observed decreases in SOD, CAT and GSH levels in TD-treated rats are pointers to oxidative stress-induced depletions of these antioxidants through the generation of reactive oxygen species. In mammals, a sophisticated antioxidant system, which includes SOD, CAT and GSH are used to maintain redox homeostasis in the liver. When the ROS is excessive, the homeostasis will be disturbed, resulting in oxidative stress, predisposing the liver to oxidative damage.^55^ Oxidative stress triggers hepatic damage by inducing irretrievable alteration of lipids, proteins and DNA contents and more importantly, modulating pathways that control normal biological functions.^56,57^ The observed increase in MDA level in TD-treated rats confirms lipid peroxidation because monitoring of MDA levels in different biological systems is an important indicator of lipid peroxidation both *in-vitro* and *in-vivo* for various health disorders.^58^ Lipid peroxidation is a chain reaction occurring during oxidative stress leading to the formation of various active compounds including propanedial and 4-hyrdoxynonenal (HNE) resulting in cellular damage.^59^ In the present study, supplementations with MT and NAC prior to treatment with TD significantly (p<0.05) decreased AST, ALP, ALT, TB and CB levels when compared to TD-treated rats (Table 3 and 4). Also, supplementation with MT and NAC prior to treatment with TD significantly (p<0.05) increased liver levels of SOD, CAT GSH whereas MDA levels were decreased in comparison to TD-treated rats (Table 5). Furthermore, histological alterations observed in the liver of TD-treated rats were ameliorated in rats supplemented with MT and NAC (Figure 4-7). Interestingly, supplementation with combined doses of MT and NAC produced significant (p<0.05) effects on AST, ALP, ALT, TB, CB, SOD, CAT, GSH and MDA levels in comparison to their individual doses (Table 3-5). The observed hepatoprotective effects of MT and NAC could be attributed to the inhibition of TD-induced hepatic oxidative stress.^60^ The best hepatoprotective effect obtained in rats’ supplemented with combined doses of MT and NAC could be attributed to the potentiation of the activity of each other through scavenging and neutralizing oxidative radicals and up-regulating the activities of some endogenous antioxidants. The ameliorative effect of NAC observed in the present study is in agreement with some authors who reported the inhibitory effect of NAC on isoniazid and rifampicin- induced oxidative liver injury in rats.^61^ Findings in this study are also consistent with studies that reported the cytoprotective effects of MT in various experimental models of acute liver injury.^62^

**Table 1:** Effects of n-acetyl cysteine and melatonin on body, relative liver weights and liver oxidative stress indices of albino Rats

**Drugs**	**Body Weight (g)**	**Relative Liver Weight (%)**	**MDA** **(U/mg protein)**	**SOD** **(U/mg protein)**	**CAT** **(U/mg protein)**	**GSH** **(µmole/mg protein)**
Control	255.3 ± 13.9	1.226 ± 0.01	0.25 ± 0.01	14.7 ± 1.36	25.2 ± 2.12	10.8 ± 1.25
NAC	260.7 ± 12.6	1.349 ± 0.06	0.24 ± 0.06	17.7 ± 1.41	27.3 ± 1.06	11.7 ± 0.79
MT	265.1 ± 14.9	1.251 ± 0.09	0.25 ± 0.08	16.2 ± 1.34	25.9 ± 2.21	11.9 ± 0.96
NAC + MT	270.5 ± 15.2	1.239 ± 0.05	0.22 ± 0.06	22.5 ± 2.28	28.3 ± 2.92	12.7 ± 1.03*

NAC= N-acetylcysteine. MT=Melatonin. n=5. Results are expressed as Mean ± SEM

**Table 2:** Effects of melatonin and n-acetylcysteine on liver function parameters of albino rats

**Drugs**	**SERUM**					**LIVER**		
	**AST (U/L)**	**ALT (U/L)**	**ALP (U/L)**	**TB (µmol/L)**	**CB (µmol/L)**	**AST (U/L)**	**ALP (U/L)**	**ALT (U/L)**
Control	34.3 ± 4.05	31.7 ± 2.42	40.0 ± 3.15	8.71 ± 1.09	3.40 ± 0.15	37.2 ± 3.15	42.7 ± 3.10	35.3 ± 2.50
NAC	33.1 ± 3.75	29.9 ± 3.72	38.7± 3.76	7.39 ± 0.11	3.01 ± 0.36	35.2 ± 2.28	40.1 ± 3.18	32.1 ± 3.91
MT	33.1 ± 3.95	31.3 ± 3.96	39.6 ± 4.06	7.33 ± 0.12	3.31 ± 0.12	36.2 ± 3.06	41.6 ± 4.97	34.1 ± 2.02
NAC+ MT	30.0 ± 2.02	29.2 ± 2.06	35.3 ± 3.65	7.23 ± 0.27	2.99 ± 0.18	35.6 ± 2.17	39.3 ± 2.91	31.3 ± 2.06

NAC= N-acetylcysteine. MT=Melatonin. n=5. Results are expressed as Mean ± SEM

**Table 3:** Effects of melatonin and n-acetylcysteine on tramadol-induced alterations in liver function parameters of albino rats

**Drugs**	**SERUM**				
	**AST (U/L)**	**ALT (U/L)**	**ALP (U/L)**	**TB (µmol/L)**	**CB (µmol/L)**
Control	34.3 ± 4.05^a^	31.7 ± 2.42^a^	40.0 ± 3.15^a^	8.71 ± 0.19^a^	3.40 ± 0.15^a^
TD	89.5 ± 6.62^b^	98.3 ± 5.42^b^	92.7 ± 8.80^b^	26.6 ± 2.13^b^	12.3 ± 0.16^b^
TD + NAC	52.1 ± 5.16^c^	57.2 ± 4.68^c^	60.3 ± 6.42^c^	14.1 ± 1.22^c^	7.01 ± 0.01^c^
TD + MT	55.3 ± 3.12^c^	60.6 ± 4.01^c^	64.2± 6.02^c^	16.3 ± 1.15^c^	7.51 ± 0.04^c^
TD+ NAC + MT	31.2 ± 2.27^a^	30.0 ± 2.95^a^	31.1± 3.85^d^	9.00 ± 0.12^a^	3.61 ± 0.06^a^

TD= Tramadol. MT= Melatonin. NAC=N-acetylcysteine. n= 5. Results are expressed as mean ± SEM. Values with different superscripts on the same column differ significantly at *p*< 0.05 ANOVA and Tukey’s multiple comparison test

**Table 4:** Effects of melatonin and n-acetylcysteine on body, liver weights and tissue levels of aminotransferases and alkaline phosphatase of tramadol-treated albino rats

**Drugs**	**WEIGHTS**		**LIVER TISSUE**		
	**Body Weight (g)**	**Relative Liver Weight (%)**	**AST (U/L)**	**ALT (U/L)**	**ALP (U/L)**
Control	255.3 ± 13.9	1.226 ± 0.01	37.2 ± 3.15^a^	35.3 ± 2.50^a^	42.7 ± 3.10^a^
TD	260.6 ± 10.1	1.237 ± 0.06	80.1 ± 7.15^b^	96.2 ± 4.10^b^	95.4 ± 7.70^b^
TD + NAC	276.1 ± 12.2	1.316 ± 0.21	50.3 ± 3.95^c^	51.7 ± 3.20 ^c^	63.2 ± 4.97^c^
TD + MT	270.3 ± 10.5	1.212 ± 0.14	53.7 ± 2.10^c^	57.2 ± 2.91^c^	65.7 ± 4.02^c^
TD+NAC+ MT	285.8 ± 14.7	1.343 ± 0.06	30.1± 1.72^d^	37.1 ± 1.96^a^	41.3 ± 3.98^a^

TD= Tramadol. MT= Melatonin. NAC=N-acetylcysteine. n= 5. Results are expressed as mean ± SEM. Values with different superscripts on the same column differ significantly at *p*< 0.05 ANOVA and Tukey’s multiple comparison test

**Table 5:** Effects of n-acetylcysteine and melatonin on liver oxidative stress indices of tramadol-treated albino rats

**Drugs**	**MDA** **(nmole/mg protein)**	**SOD** **(U/mg protein)**	**CAT** **(U/mg protein)**	**GSH** **(µmole/mg protein)**
Control TD	0.25 + 0.01^a^ 1.15 ± 0.06^b^	14.7± 1.36^a^ 3.59 ± 0.27^b^	25.2 ± 2.12^a^ 6.51 ± 0.13^b^	10.8 ± 1.25^a^ 3.21 ± 0.08^b^
TD +NAC	0.58 ± 0.01^c^	7.15 ± 0.16^c^	13.1 ± 0.29^c^	5.98 ± 0.16^c^
TD+ MT	0.62 ± 0.03^c^	6.95 ± 0.01^c^	11.3 ± 0.31^c^	5.10 ± 0.13^c^
TD+NAC+MT	0.37 ± 0.05^d^	12.01 ± 0.26^a^	23.0 ± 1.01^a^	9.98 ± 0.15^a^

TD= Tramadol. MT= Melatonin. NAC=N-acetylcysteine. n= 5. Results are expressed as mean ± SEM. Values with different superscripts on the same column differ significantly at *p*< 0.05 ANOVA and Tukey’s multiple comparison test

MT and NAC are free radical scavengers and neutralizers which can inhibit oxidative stress and lipid peroxidation.^63,64^ Metabolites of MT, including the major hepatic metabolite 6-hydroxymelatonin, as well as N-acetyl-N-formyl-5-methoxykynuramine and N-acetyl-5-methoxykynuramine have been shown to detoxify radicals.^65,66^ In addition, MT may down-regulate pro-oxidant enzymes like nitric oxide synthase (NOS) and lipoxygenases, thus reducing the formation of nitric oxide (NO), superoxide anions, and subsequently peroxynitrite anions.^67,68^ MT and NAC can stabilize membranes and increase their resistance toward free radical attack.^69,70^ Furthermore, MT and NAC can stimulate the production or regeneration of antioxidants including SOD, CAT and GSH. Studies have shown that NAC and MT experimentally enhanced intracellular glutathione level by stimulating the rate-limiting enzyme required for it synthesis.^71,72^ Also, NAC has been reported to prevent xenobiotic-induced hepatotoxicity by inhibiting the hepatic depletion of GSH and up-regulating its activity. Hepatoprotective effect of NAC can also occur through maintaining –SH groups of enzymes and membrane proteins in their reduced state.^73^

## Conclusion

The findings in the present study showed the potential of melatonin and n-acetylcysteine as remedies for hepatotoxicity associated with the abuse or clinical use of tramadol.

## Acknowledgments

The authors would like to appreciate the technical assistance of Mr Eze Iheukwumere of the Faculty of Pharmacy, Madonna University, Elele, Rivers State.

## Ethical Issues

All rats used for this study were handled in accordance with Directive 2010/63/EU of the European Parliament and the Council on the protection of animals used for scientific purposes.

## Conflict of Interest

The authors declare no conflict of interests in the authorship and the publication of this research.
